# Supplementary material for: Chromothripsis during telomere crisis is independent of NHEJ, and consistent with a replicative origin
Source: Genome Res. 2019 May;29(5):737–49. doi: 10.1101/gr.240705.118 (PMC6499312; doi:10.1101/gr.240705.118)
Supplement: Supplemental Material [file supp_gr.240705.118_Supplemental_file_1.zip › contigs/annotated_contigs/DB79/contig.3.DB79_length_444_mean_cov_4.05405405405.docx]

**DB79_length_444_mean_cov_4.05405405405**

```
TGGGGAGGTCGGGATGCCTGGGATCCCTTCCTGGAGAGGCAGGCTGTCTCTGGAAAAAGCCTCCATTGCCCACCCGCCAGGCGGAAAGT
 >chr9:132998450-132998785 + E=2e-162
CACCCTGTTCCCAGCGCGGTTTCAGCATTTAATTTTAAGGGAGCTAAGGAAGCGCGGCGCGCCCGCTGGGGGTGGTAAACAGCGAACAC

ACCTGGGGGGTGCCACCCCCCCGGACGGGTGGTCCGGAGGGAGGCTGGAGCGGGGAGGCGAGGAGGGGGCTGTGAGTCCTCAGAGGCCC

TGGGCCACCACATTTCTGGCAGCGTTTCCCAGACACCCCTCTGCTAGGCCATCCCTGGATAGCAAGTG|TTTTTTGCCAACCTGATTGG
                                                                      >chr9:98905218-989
CAAACAACTGAGTCTCATTATTGTTGTAATAAACATTCTCTGCAGTACTAGTGATGTTGGGCATATATTTAAATGTTTGACAATCATTT
05328 + E=3e-54
T|ACTCCTGAACTTAAAATAAAAGTTAAAAATAAAAGTTAATTTAATTTAAAAAATAAAAATATTATAAAAAAAGTATAAAAAATTATA
 >chr4:70282241-70282690 - E=2e-227
TGGAGTCATTGTAATTCTAGTGTAATAAAACAGTAACTATTTAGTAATGACCTATATGTTTGTTTTATGTATTTTATATTAGTCTTCAT

CTCCAAATTTAATTGTAATGTATTCATTTATTTTTGAATAATATGAGAAGTAGTTTAAATATATCTACTTGTCGGTAGGTTATTACATT

AGAGCCTGTTGTCCTTTGATTGACAAAATAGCAAGTCTGTAAAATAAGCTACTTATAATAAATAAATAAATGCTGCCAAGGTGTTATGT

ATAACTTCATTGGAATTACAAATTATCAAGCACTACTTCCAAGAAAAATTTCAAGTGACAATTTTAGACACAAATTCAGAATATTACCG
```
